# Case report: Pediatric low-grade gliomas: a fine balance between treatment options, timing of therapy, symptom management and quality of life

**DOI:** 10.3389/fonc.2024.1366251

**Published:** 2024-06-07

**Authors:** Nicolette Joh-Carnella, Glenn Bauman, Torunn I. Yock, Shayna Zelcer, Sabin Youkhanna, Chantel Cacciotti

**Affiliations:** ^1^ Schulich School of Medicine and Dentistry, Western University, London, ON, Canada; ^2^ Division of Radiation Oncology, Department of Oncology, London Health Sciences Centre & Western University, London, ON, Canada; ^3^ Department of Pediatric Radiation Oncology, Massachusetts General Hospital, Boston, MA, United States; ^4^ Division of Hematology/Oncology, Department of Pediatrics, London Health Sciences Centre & Western University, London, ON, Canada; ^5^ Department Radiation Oncology, London Regional Cancer Centre, London, ON, Canada

**Keywords:** pediatric low-grade glioma, pilocytic astrocytoma, proton radiation, chemotherapy, case report

## Abstract

**Introduction:**

Pediatric low-grade gliomas (pLGG) are the most common brain tumor in children and encompass a wide range of histologies. Treatment may pose challenges, especially in those incompletely resected or those with multiple recurrence or progression.

**Case description:**

We report the clinical course of a girl diagnosed with pilocytic astrocytoma and profound hydrocephalus at age 12 years treated with subtotal resection, vinblastine chemotherapy, and focal proton radiotherapy. After radiotherapy the tumor increased in enhancement temporarily with subsequent resolution consistent with pseudoprogression. Despite improvement in imaging and radiographic local control, the patient continues to have challenges with headaches, visual and auditory concerns, stroke-like symptoms, and poor quality of life.

**Conclusion:**

pLGG have excellent long-term survival; thus, treatments should focus on maintaining disease control and limiting long-term toxicities. Various treatment options exist including surgery, chemotherapy, targeted agents, and radiation therapy. Given the morbidity associated with pLGG, individualized treatment approaches are necessary, with a multi-disciplinary approach to care focused on minimizing treatment side effects, and promoting optimal quality of life for patients.

## 1 Introduction

Pediatric low-grade gliomas (pLGG) are one of the most common childhood brain tumors, accounting for about one-third of such tumors. The clinical behavior varies, but pLGG are indolent and carry a low risk of malignant transformation, with a 5-year overall survival (OS) as high as 97%, and 10- and 20-year OS around 90% (1, 2). Progression-free survival (PFS) is inferior, especially in those with residual tumor, where PFS has been documented as high as 45%-65% (3). These tumors can occur in deep locations such as the brainstem and suprasellar area; treatments and tumoral location may result in considerable morbidity, including vision loss, functional decline, endocrine dysfunction, motor disability, neurocognitive difficulties, and reduced quality of life (QoL). Management is aimed at long-term tumor control while minimizing tumor- and treatment-related morbidity and maintaining QoL (4, 5).

Gross total resection is the preferred treatment for pLGG when feasible (6). Unresectable tumors or those that progress require adjuvant treatment with chemotherapy, targeted agents, and/or radiation therapy (6, 7). With the emergence of molecular diagnostics suggesting most pLGG upregulate the RAS mitogen-activated protein kinase (RAS/MAPK) pathway, targeted therapies are a promising treatment option (4, 8). Early studies offer optimistic results, but long-term side effects are yet unknown; should current clinical trials report efficacious and safe treatment of pLGG, this modality has the potential to become first-line treatment of pLGG (9) Chemotherapy remains a front-line adjuvant therapy for children with progressive or unresectable pLGG. Typically monotherapy with vinblastine or carboplatin or combination treatment with carboplatin and vincristine or thioguanine, procarbazine, lomustine/CCNU and vincristine (TPCV) are utilized (10, 11). Chemotherapy is associated with a 3-year PFS of 50–80% (6), and side effects are taken into consideration (7).

Radiation therapy has become less favored as first-line therapy in young patients (i.e., those under 10 years old) due to its potential long-term effects, including neurocognitive and endocrine dysfunction as well as risk of second malignancy (7). Although developments in radiation technology, such as imaged guided intensity modulated photon and proton beam radiation, can significantly reduce side effects (12), the high OS associated with pLGG, alternative treatment options, and low likelihood of malignant transformation have resulted in less frequent use. Radiation therapy may serve as a reasonable option in older pLGG patients, those with symptomatic progression, and/or those with progressive disease despite systemic therapy.

The timing of various treatments and their potential side effects relative to morbidity associated with tumor progression and cumulative effects of other treatment options need to be carefully considered (13, 14). Herein, we report the multi-year clinical course of a 12-year-old female diagnosed with a pLGG and ultimately treated with subtotal resection, vinblastine chemotherapy, and focal proton radiotherapy. While our patient’s disease was adequately treated with this combination of therapy, her QoL has significantly suffered as she continues to experience effects of the tumor itself as well as its associated treatment.

## 2 Case description

**Figure 1:**
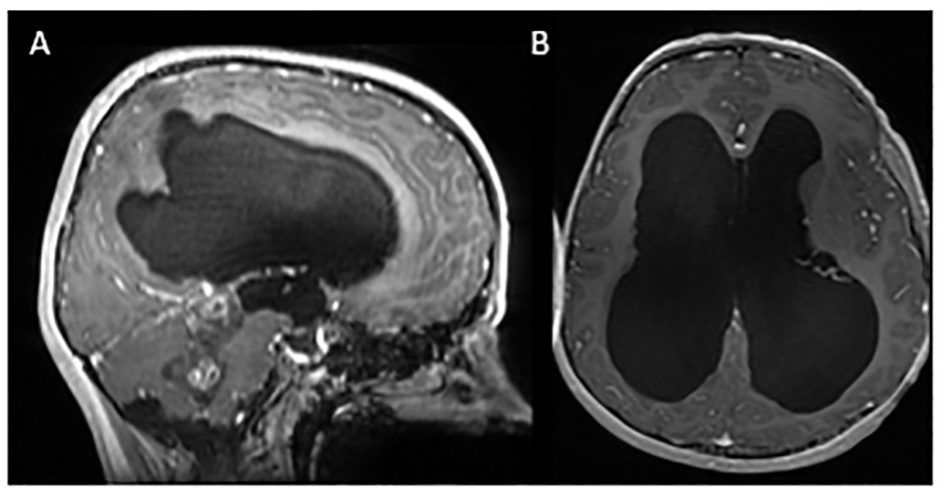
Initial MRI imaging demonstrating mass and associated hydrocephalus. Sagittal **(A)** and axial **(B)** post contrast images demonstrating fourth ventricular mass with associated hydrocephalus.

**Figure 2:**
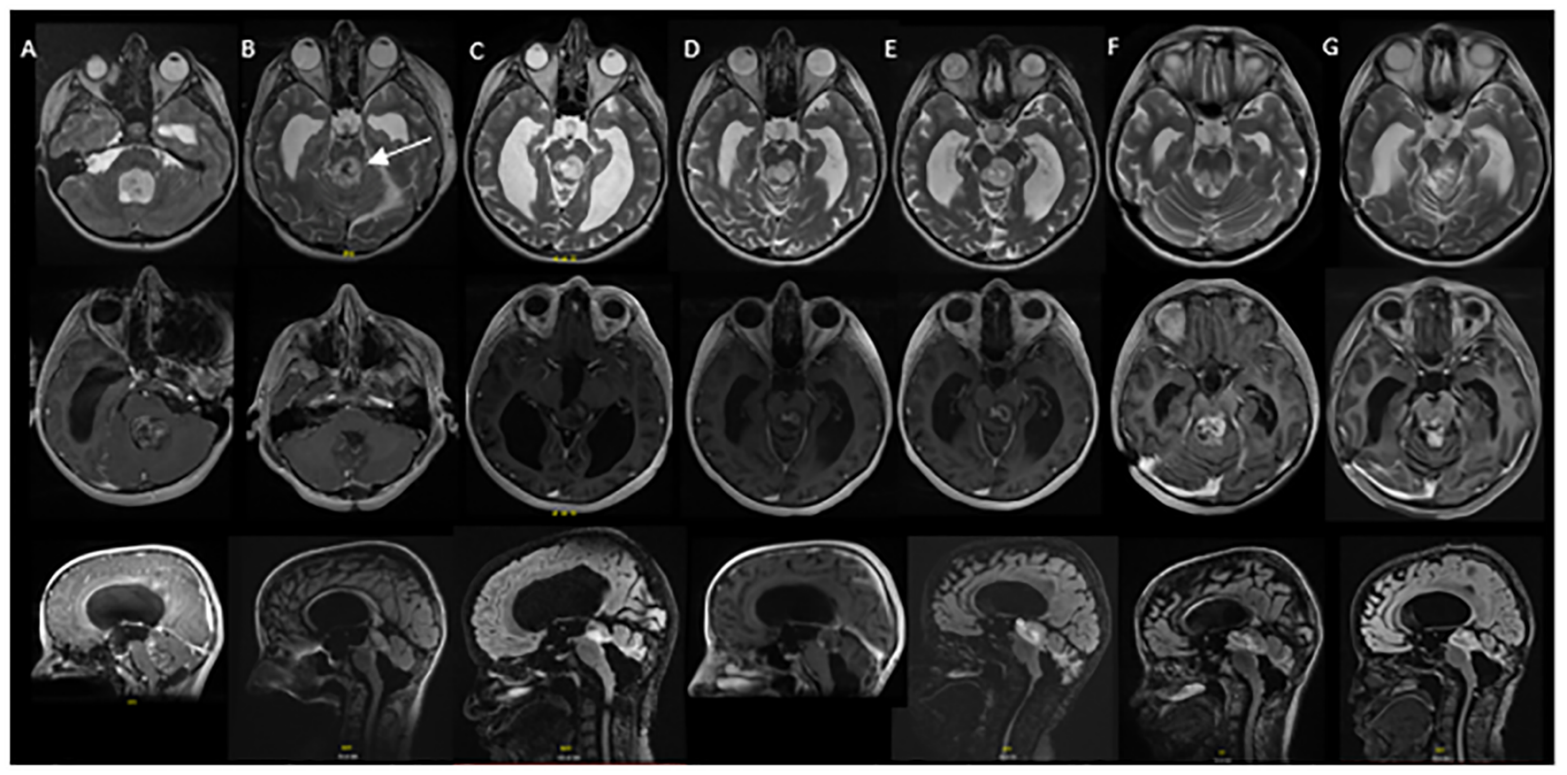
Serial MRI imaging demonstrating tumor changes over time. Axial high-resolution images on top panel, axial T1 post contrast images in middle panel and sagittal T1 post contrast images in bottom panel [**(B)** and **(C)** bottom are sagittal FLAIR images]. **(A)** Mass noted within the fourth ventricle resulting in supratentorial hydrocephalus and trans ependymal edema. **(B)** Post-operative MRI demonstrating residual tumor in the midbrain (arrow) and pons as well as roof of fourth ventricle (1 week post initial MRI). **(C)** Local tumor progression with enlargement of nodular component of dorsal midbrain mass and increased enhancement (64 months from initial diagnosis). **(D)** Completion of vinblastine chemotherapy, tumor stable on imaging (84 months from diagnosis). **(E)** Further tumor progression with increase in size of posterior midbrain mass (92 months from diagnosis). **(F)** Following radiation therapy, tumor appears stable in size although increased enhancement of the tumor was noted in the pons, midbrain and subthalamic regions (105 months from diagnosis). **(G)** Tumor stable on most recent evaluation (152 months from initial diagnosis).

A 12-year-old previously healthy female presented with a 2–3-month history of intermittent headaches, dizziness, emesis, and unsteady gait. Neurological assessment revealed slow and deliberate speech, papilledema, decreased lower extremity tone, bilateral dysmetria, and ataxia. MRI brain revealed a heterogeneously enhancing mass in the fourth ventricle with obstructive hydrocephalus (**Figures 1**, **2A**). The patient’s treatment included endoscopic third ventriculostomy and subtotal tumor resection (**Figure 2B**). Surgical management of pediatric CNS tumors is specialized, thus centralization of care at large pediatric centers is imperative. Her post-operative course was complicated by cerebral salt wasting, ophthalmoplegia, and diplopia. Pathology was consistent with a pilocytic astrocytoma, WHO grade I; molecular testing, now considered standard of care, was not performed.

**Figure 3:**
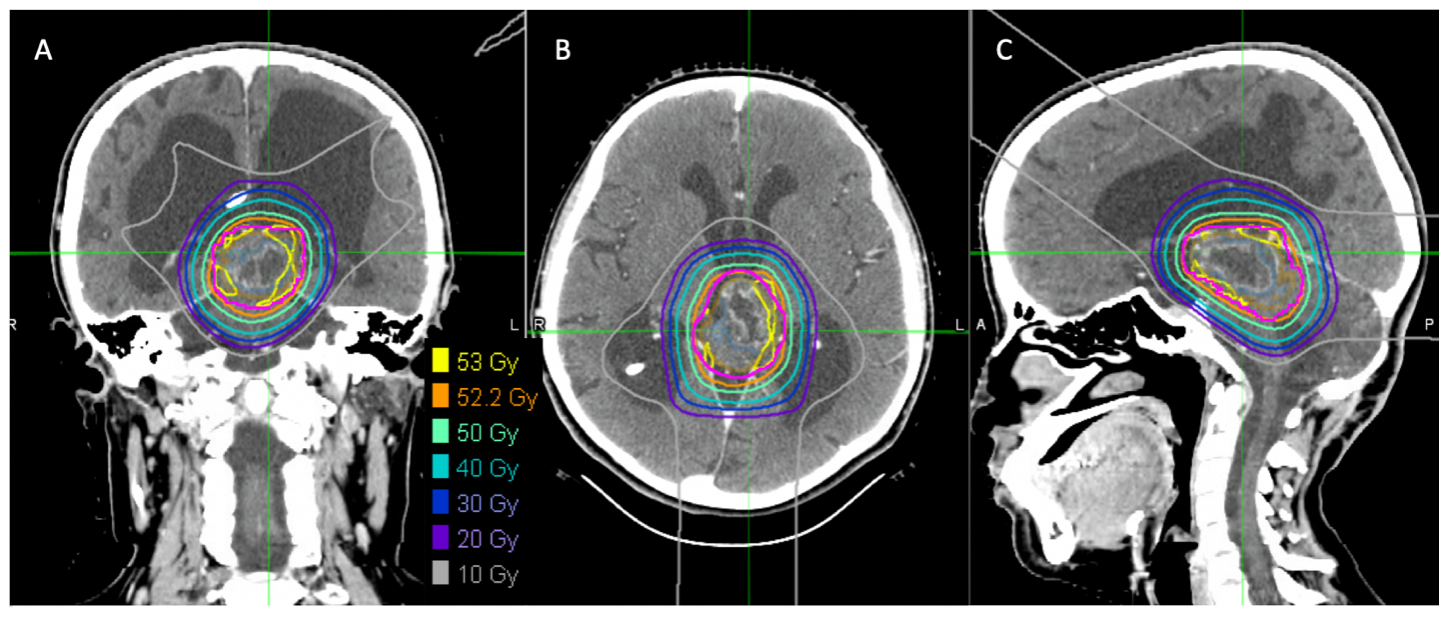
Proton radiation plan demonstrating doses administered. Coronal **(A)**, axial **(B)** and sagittal **(C)** images demonstrating proton radiation plan and doses administered.

Local tumor progression was identified on surveillance imaging 5 years after initial diagnosis (**Figure 2C**). The patient experienced clinical progression with right-sided hearing loss. Given the tumor location, additional surgery was not feasible; she was started on vinblastine chemotherapy. Dose reduction (4mg/m2/dose) was required secondary to intolerance, specifically nausea, peripheral neuropathy, and myelosuppression. She completed a 70-week course of chemotherapy as planned, with subsequent tumor stability (**Figure 2D**). Throughout treatment the patient struggled with episodic headaches, ataxia, diplopia, and neuropathic pain. She completed high school but was unable to pursue further education given her functional status. Approximately 8 months post chemotherapy, the patient developed further clinical and radiographic progression with vomiting and headaches (**Figure 2E**). At this time, a right ventriculoperitoneal (VP) shunt was inserted which improved performance status. Subsequent treatment options were discussed and ultimately the patient proceeded with focal proton beam radiation (5220cGy/29 fractions) (**Figure 3**). At presentation and throughout her treatment, she was followed by allied health professionals. Medications were used to help manage pain, neuropathy, tinnitus, headaches, and nausea.

Five months following completion of radiation, the patient developed worsening headaches, fatigue, unsteadiness, dizziness, word-finding difficulties, auditory symptoms, and visual symptoms, raising concern for a stroke. Neuro-imaging ruled out a stroke and demonstrated stability in tumor size, with new T2 changes and enhancement surrounding the tumor (**Figure 2F**). Differential included post-radiation effects, pseudoprogression, or true tumor progression. Dexamethasone was initiated, but due to myopathy was discontinued.

Ongoing surveillance over the subsequent 5 years demonstrated tumor stability and eventual improvement of the peritumoral T2 changes and enhancement (**Figure 2G**) suggesting the initial changes were related to pseudoprogression. The patient continued to endorse headaches, diplopia, hearing impairment, tinnitus, ataxia, and fatigue. Despite combination treatment that ultimately achieved durable tumor control, her long term QoL has been adversely affected since diagnosis.

## 3 Discussion

pLGG are indolent tumors described as a chronic progressive disease that may require multiple treatment modalities. The mainstay of therapy is complete resection, when feasible (5, 6). In those with residual disease, timing of adjuvant therapy is controversial, with some suggesting a “watch and wait” approach as a quiescent period is possible and others considering more immediate treatment (3, 6). The plethora of treatment options and their associated toxicity weighed against the potential complication of tumor progression need to be taken into consideration in the management of these patients.

LGG of childhood have been recognized as distinct from those arising in older adolescents and adults (8, 15). In contrast to adult LGG, pLGG rarely undergo malignant transformation, although the precise frequency of this transformation in the absence of radiotherapy in the management of pLGG remains unknown as radiotherapy is often used at progression and repeat biopsy is seldom performed (15). In adult patients with shorter life expectancies and whose tumors are typically more aggressive than children’s, early irradiation remains standard practice (16).

Historically photon radiation had been used in pLGG, in both up-front and salvage therapy, with 5-year PFS and OS of 87% and 99%, respectively (17). Radiation, albeit an effective treatment, is not without side effects, some of which greatly impact QoL (14, 18–20). Photon radiotherapy is associated with long-term side effects including neurocognitive decline, behavioral changes, increased risk of stroke, neuroendocrine deficiencies, vascular damage, growth abnormalities, and increased risk of second malignancy (**Table 1**) (30–34). Neuropsychiatric impacts of brain radiation need to be further explored especially in the modern era of radiotherapy.

**Table 1:** pLGG treated with radiation therapy in the literature.

Author/Year	Mean age at RT (year; range)	Pathology	RT modality	Median RT dose (range) Gy	Patients who received chemotherapy prior to RT	Prior surgical resection/biopsy	Outcome	Toxicity reported
Rodrigues et al., 2021 (21)	9.22 years	Astrocytoma NOS (n=249, 68%) Pilocytic astrocytoma (n=64, 17.5%) Diffuse astrocytoma (n=22, 6%) Oligodendroglioma (n=5, 1.4%) Glioma NOS (n=18, 4.9%) Mixed glioma (n=8, 2.2%)	EBRT		34/366 (9.3%)	Surgery (n=248, 67.8%)	No survival assessment in study	Secondary neoplasm 7.4% in RT treated group
Indelicato et al., 2019 (20)	10.2 (2–21) years 48.6% (n=36) of the patients were <30 years old	WHO grade I: (n=122, 70%) WHO grade II: (n=52, 30%)	Proton	129 treated with 54Gy and 45 treated with <54 Gy	74/174 (43%) 1 prior regimen (n=29, 17%), 2 prior regimens (n=23, 13%). 3+ prior regimen (n=22, 13%)	No prior surgery (n=22, 13%) STR/biopsy (n=147, 84%) GTR (n=5, 3%)	5-year PFS and OS 84% and 92%, respectively	Reduced local control in brainstem/spinal cord tumor (62% vs 90% other locations) and in those that received dose <54 Gy (67% in <54Gy vs 91%) Nausea or vomiting (12.6%) New central hormone deficiency (22%) Pseudo progression (32%) Significant toxicity in 4% of patients; brainstem necrosis requiring corticosteroids (n=2), symptomatic vasculopathy (n=2), radiation retinopathy (n = 1), epilepsy (n = 1), and death from radiation-induced high-grade glioma (n = 1).
Ludmir et al., 2019 (22)	10 (1–17.6) years	WHO grade I (n=62, 75%) WHO grade II (n=21, 25%)	IMRT (n=32, 39%) Proton (n=51, 61%)	50.4 (45–59.4) Gy	32/83 (39%)	Biopsy (n=42, 51%) STR (n=37, 45%) GTR (n=4, 5%)	Improved local control with proton RT (HR 0.34, 95% CI: 0.10–1.18, p=0.099)	Pseudo progression (n=31, 37%); 8/32 (25%) IMRT patients and 23/51 (45%) proton (p=0.048). Higher doses of RT (>50.4Gy) were more likely to have pseudo progression (p=0.016)
Cherlow et al., 2019 (23)	13.6 (3–21) years *(median)*	Pilocytic astrocytoma (n=66; 78%) Diffuse astrocytoma (n=12, 14%) LGG NOS (n=2, 2%) LGG oligodendroglioma (n=1, 1%)	IMRT (n=60, 71%) 3D-CRT (n=25; 29%)	54 Gy	36/85 (42%)		PFS (5-year) 71% OS (5-year) 93%	Tumor necrosis (n=1) Acute visual loss reversed with steroids (n=1) Acute diplopia reversed with steroids (n=1)
Mannina et al., 2016 (24)	10.9 (4–20) years	WHO grade I (n=15, 100%)	Proton	54 (50.4–59.4) Gy	9/15 (60%)	Biopsy only: (n=5, 33%) ≥ 1 subtotal resection: (n=10, 67%) 2 STR (n=3, 30%)	5-year OS and intervention free survival 93% and 73%, respectively	Pseudo progression (20%) Secondary malignancy, ALL (n=1), radio necrosis (n=1)
Raikar et al., 2014 (25)	9.4 years	WHO grade I (n=10, 59%) WHO grade II (n=7, 41%)	Conformal RT (n=13, 76%) CyberKnife (n=2, 12%) Gamma Knife (n=2, 12%)	50–54 Gy (CRT) 14–16Gy (GammaKnife) 21–26Gy (CyberKnife)	13/17 (76%) 1 prior regimen (n=7, 54%), 2 prior regimens (n=4, 31%), 3 prior regimens (n=1, 8%), 4 prior regimens (n=1, 8%)	Biopsy (n=7, 41%) STR (n=10, 59%) GTR (n=0)	PFS (3-year) OS (3 and 10-year) 100%	
Greenberger et al., 2014 (18)	11.0 (2.7–21.5) years	WHO grade I: (n=19, 59.4%) WHO grade II: (n=6, 18.8%) low grade (not specified) (n=2, 6.3%), no pathology: (n=5, 15.6%)	Proton	52.2 (48.6–54) Gy	16/32 (50%) One prior regimen (n=6, 18.8%) 2 prior (n=7, 21.9%) 3 prior (n=3, 9.4%) none (n=16,50%)	No prior surgery: (n=5, 15.6%) Biopsy only: (n=6, 18.7%) 1 prior resection: (n=17, 53.1%) 2 or more resections: (n=4, 12.5%)	6-year PFS 89.7%, 8-year PFS 82.8%; OS (8-year) 100%	Decline in neurocognitive outcome in children < 7 years in age and those with higher doses to left temporal lobe/hippocampus. Higher risk of endocrinopathy in patients with mean dose of ≥40 Gy to hypothalamus, pituitary, or optic chiasm Moya moya (n=2)
Paulino et al., 2013 (26)	10 (1–17) years *(median)*	WHO grade I (n=32, 82%) WHO grade II (n=7; 18%)	IMRT	50.4 Gy (45–54Gy)	10/39 (25.6%)	STR (n=19; 48.7%)	PFS (8-year) 78.2%, OS (8-year) 93.7%	Age at time of RT was significant for PFS, with more disease progression observed in patients ≤5 years of age at time of IMRT. Moya moya (n=1) Children with centrally located tumor more likely to develop endocrine abnormalities compared to hemispheric or posterior fossa tumors, hormone deficiency (n=10)
Merchant et al., 2009 (27)	9.7 (2.2–19.8) years	WHO grade I (n=67, 86%) WHO grade II (n=11, 14%)	IMRT (n=3, 4%) 3D-CRT (n=75, 96%)	50.4 (one patient with OPG), otherwise 54Gy in all others	25/78 (32%)	Biopsy (n=30, 38%) STR (n=35, 45%) No prior surgery (n=13, 17%)	EFS 87.4% (5-year), 74.3% (10-year) OS 98.5% (5-year), 95.8% (10-year)	Vasculopathy (n=5); younger children <5yo were at greatest risk Second malignancy (n=1) Younger age associated with more marked decline in cognitive scores with most marked decline in <5yo Thyroid hormone and GH deficiencies (10-year cumulative incidence), 64% and 48.9%, respectively
Marcus et al., 2005 (28)	9 (2–26) years	WHO grade I (n=35, 70%) WHO grade II (n=15, 30%)	SRT	Mean 52.2 (50.4–58) Gy	12/50	STR (n=38, 76%)	PFS (5-year) 82.5% (8-year) 65% OS 97.8% at 5-years, 82% at 8 years	Transformation to higher grade tumor, anaplastic astrocytoma (n=2) RT induced PNET (n=1) Moya-moya (n=4) No significant acute toxicity attributable to SRT
Hug et al., 2002 (29)	8.7 (2–18) years	Diffuse low grade astrocytoma (n=9, 33%) JPA (n=14, 52%), no path (n=4, 19%)	Proton	Mean 55.2 (50.4–63)	No comment on prior therapy	STR/biopsy (n=25, 92%) GTR, but residual enhancement (n=1, 4%) GTR (complete radiographic resection) (n=1, 4%)	At mean follow up 3.3 years 6/27 patients local failure, 4/27 died	Transformation to high grade GBM (n=1) New onset hypopituitarism (n=4) Moya moya (n=1)

EBRT, external beam radiation therapy; IMRT, Intensity-modulated radiation therapy; SRT, Stereotactic radiotherapy; WHO, World Health Organization; GTR, Gross total resection; STR, Sub total resection; RT, radiation; PFS, Progression free survival; OS, Overall survival.

Chemotherapy is an option in progressive or incompletely resected pLGG as a means to delay or avoid radiotherapy (6, 10, 11). The combination of carboplatin and vincristine is associated with a PFS of 68% (12). TPCV is similarly effective, but is associated with a risk of second malignancy and infertility (35). Some patients receive multiple lines of chemotherapy for recurrent disease, and their QoL and risk of treatment toxicity remains a concern.

In those patients that exhaust other therapy options, radiation becomes a treatment consideration. Newer radiation techniques, such as intensity modulated, image guided photon or proton beam radiation offer treatment with the potential of reducing radiation-associated toxicity (**Table 1**) (20, 30). Proton beam radiation, which our patient received, allows for improved sparing of normal brain tissue (20, 22, 30). Although data are limited, initial studies report that proton therapy is effective in pLGG at maintaining high PFS and OS while reducing radiation-induced side effects (18, 20, 30). Some series have suggested a higher risk of pseuodoprogression following proton beam radiotherapy compared to photon radiotherapy; a recent systematic review suggested no difference (22, 36, 37). In our patient, there was radiographic as well as clinical deterioration following radiotherapy. While the imaging changes resolved, the clinical symptoms persisted. Although most instances of pseudoprogression are diagnosed on imaging alone, clinical symptom progression is possible (22, 38, 39).

Stereotactic radiation therapy (SRT), another highly conformal radiation approach, has also been shown to be effective in the management of pLGG (28). Similar to proton radiation therapy, the goal of SRT is to minimize the amount of normal tissue irradiated without compromising tumor management (28). Second malignant neoplasm (SMN) specifically high-grade glioma, although rare, serves as a possible late effect of radiation therapy. Chemotherapy, specifically TPCV, is also associated with a risk of SMN, specifically leukemia, and thus tends to be a less favored chemotherapy regimen (20, 35, 40). Furthermore, children with neurofibromatosis type 1 (NF-1) who are at increased risk of pLGG, also have an increased risk of SMN with one study documenting a relative risk of 3.04 of SMN after radiation therapy (41).

Although not applicable in our case, clinicians considering radiation therapy should be aware of the well-documented cranial radiation-induced vascular complications (27, 42–45). The pathophysiology of this is complex; it involves endothelial loss and ultimately results in vascular damage and abnormal endothelial proliferation involving the upregulation of pro-inflammatory and hypoxia-related genes (42, 46). Certain factors including tumor location (i.e., circle of Willis), younger age at time of irradiation, NF-1, treatment with an alkylating chemotherapy agent, and higher doses of radiation increase the risk for cerebrovascular complications (43–45, 47–50).

The emergence of molecular diagnosis allowing for targeted therapy is changing the landscape of pLGG management. These tumors frequently have somatic driver alterations that result in MAPK pathway activation (8). Initial clinical trials offer promising results but more data are needed to evaluate long-term efficacy and side effects (9). Notably, molecular diagnostics were not available for our patient at the time of presentation for consideration of radiotherapy; molecular characterization of disease is done routinely in cases of pLGG.

In most cases of residual or unresectable disease, systemic therapy is not curative and serves primarily as a radiotherapy deferral strategy particularly among younger patients who are at highest risk of long-term deficits. That said, an “avoid radiotherapy until absolutely necessary” strategy may not serve all patients well as radiation will not reverse pre-existing toxicity deficits incurred through successive rounds of tumor progression and interventions. It is unknown if proton therapy was introduced earlier in her care (i.e. at the time of first progression after surgery when the patient was age 17), in aggregate would have had a more favorable longer term therapeutic profile than the patient experienced.

QoL is an important aspect of clinical care that encompasses various aspects of a person’s well-being and reflects satisfaction with life (51, 52). As a broad term it tends to be defined as an individual’s sense of well-being and ability to participate in and enjoy life. QoL includes physical, psychological and functional status, as well as social and emotional wellbeing (53–57). There are various standardized questionnaire that focus on general symptoms and patients ability to function, some of these include questions pertaining to difficulties with symptoms such as headaches, anorexia, nausea, seizures, sleep disturbances, mood, social interactions or isolation, motor difficulties, cognitive abilities and one’s ability to perform basic activities of daily living (57–60). QoL is impacted by patient specific factors, tumor location, treatment and side effects from the treatment and patients overall experience (53). In our case, no formal tool was used to assess QoL, instead subjective QoL was reported based on the patient’s symptoms.

## 4 Conclusion

As a chronic disease, pLGG tend to require multiple modalities of therapy. Patients’ QoL can be significantly impacted both by symptoms of tumor progression as well as treatment side effects. The heterogenous nature of this disease and varying clinical course results in challenges in management. The treatment-related effects should be considered. In some circumstances, the cumulative effects of multiple lines of surgery and systemic therapy in addition to the tumoral’s negative impact on function at diagnosis and at progression likely play a significant role in patients’ poor health-related QoL outcomes. For some patients, earlier intervention with radiotherapy (accepting potential longer-term toxicity of this modality) with its associated durable tumor control might be the appropriate strategy to secure optimal long-term QoL as even the most advanced technical delivery of radiation typically cannot recover function that has been lost. Overall, these patients require individualized approaches to management with a focus on multi-disciplinary team involvement to reduced treatment-associated side effects, and promote QoL.

## 5 Patient perspective

For the past 15 years, I have struggled through surgery, chemotherapy, and radiotherapy and all of the side effects that come with all of those treatments. None of them were easy and there is no one path that I favor more than the others, they are all equally difficult to endure. Separately I don’t believe they were as helpful as they were when combined altogether. I am thankful to be able to receive all of these important treatments and my long survival. Though I’ve been left disabled after everything, I am thankful to be alive and to be able to enjoy my life with my family. I am also thankful to all of the very knowledgeable doctors for each part that they have played in my treatment. It has been a painful and arduous journey that I’ve been through and it has been full of loss, and though my life is very different than that of the average person, that doesn’t mean it’s not enjoyable or fulfilling. Life goes on, and it doesn’t have to go on the same way for everyone to be considered a good life.

## Data availability statement

The original contributions presented in the study are included in the article/supplementary material. Further inquiries can be directed to the corresponding author.

## Ethics statement

Written informed consent was obtained from the individual(s) for the publication of any potentially identifiable images or data included in this article.

## Author contributions

NJ-C: Writing – original draft. GB: Conceptualization, Writing – review & editing. TY: Writing – review & editing. SZ: Writing – review & editing. SY: Writing – original draft. CC: Conceptualization, Writing – original draft, Supervision.

## References

[1] Krishnatry R Guerreiro Stucklin AS Pole JD Mistry M Fried I . Clinical and treatment factors determining long-term outcomes for adult survivors of childhood low-grade glioma: A population-based study. Cancer. (2016) 122:1261–9. doi: 10.1002/cncr.29907 26970559

[2] Bandopadhayay P Bergthold G London WB Goumnerova LC Morales La Madrid A Marcus KJ . Long-term outcome of 4,040 children diagnosed with pediatric low-grade gliomas: an analysis of the Surveillance Epidemiology and End Results (SEER) database. Pediatr Blood Cancer. (2014) 61:1173–9. doi: 10.1002/pbc.24958 PMC4657506 24482038

[3] Shaw EG Wisoff JH . Prospective clinical trials of intracranial low-grade glioma in adults and children. Neuro Oncol. (2003) 5:153–60. doi: 10.1215/S1152851702000601 PMC1920689 12816721

[4] Ryall S Tabori U Hawkins C . Pediatric low-grade glioma in the era of molecular diagnostics. Acta Neuropathol Commun. (2020) 8:30. doi: 10.1186/s40478-020-00902-z 32164789 PMC7066826

[5] Wisoff JH Sanford RA Heier LA Sposto R Burger PC Yates AJ . Primary neurosurgery for pediatric low-grade gliomas: a prospective multi-institutional study from the Children's Oncology Group. Neurosurgery. (2011) 68:1548–54. doi: 10.1227/NEU.0b013e318214a66e 21368693

[6] de Blank P Bandopadhayay P Haas-Kogan D Fouladi M Fangusaro J . Management of pediatric low-grade glioma. Curr Opin Pediatr. (2019) 31:21–7. doi: 10.1097/MOP.0000000000000717 PMC6664811 30531227

[7] Rosca L Robert-Boire V Delisle JF Samson Y Perreault S . Carboplatin and vincristine neurotoxicity in the treatment of pediatric low-grade gliomas. Pediatr Blood Cancer. (2018) 65:e27351. doi: 10.1002/pbc.27351 30014595

[8] Ryall S Zapotocky M Fukuoka K Nobre L Guerreiro Stucklin A Bennett J . Integrated molecular and clinical analysis of 1,000 pediatric low-grade gliomas. Cancer Cell. (2020) 37:569–83.e5. doi: 10.1016/j.ccell.2020.03.011 32289278 PMC7169997

[9] Manoharan N Liu KX Mueller S Haas-Kogan DA Bandopadhayay P . Pediatric low-grade glioma: Targeted therapeutics and clinical trials in the molecular era. Neoplasia. (2023) 36:100857. doi: 10.1016/j.neo.2022.100857 36566593 PMC9803951

[10] Lassaletta A Scheinemann K Zelcer SM Hukin J Wilson BA Jabado N . Phase II weekly vinblastine for chemotherapy-naïve children with progressive low-grade glioma: A canadian pediatric brain tumor consortium study. J Clin Oncol. (2016) 34:3537–43. doi: 10.1200/JCO.2016.68.1585 27573663

[11] Packer RJ Lange B Ater J Nicholson HS Allen J Walker R . Carboplatin and vincristine for recurrent and newly diagnosed low-grade gliomas of childhood. J Clin Oncol. (1993) 11:850–6. doi: 10.1200/JCO.1993.11.5.850 8487049

[12] Packer RJ Ater J Allen J Phillips P Geyer R Nicholson HS . Carboplatin and vincristine chemotherapy for children with newly diagnosed progressive low-grade gliomas. J Neurosurg. (1997) 86:747–54. doi: 10.3171/jns.1997.86.5.0747 9126887

[13] Sait SF Giantini-Larsen AM Tringale KR Souweidane MM Karajannis MA . Treatment of pediatric low-grade gliomas. Curr Neurol Neurosci Rep. (2023) 23:185–99. doi: 10.1007/s11910-023-01257-3 PMC10121885 36881254

[14] Grippin AJ McGovern SL . Proton therapy for pediatric diencephalic tumors. Front Oncol. (2023) 13:1123082. doi: 10.3389/fonc.2023.1123082 37213290 PMC10196353

[15] Rubin JB Finlay JL . Pediatric low-grade gliomas: a brave new world. Neuro Oncol. (2018) 20:149–50. doi: 10.1093/neuonc/nox221 PMC5777489 29365202

[16] Greuter L Guzman R Soleman J . Pediatric and adult low-grade gliomas: where do the differences lie? Children (Basel). (2021) 8(11):1075. doi: 10.3390/children8111075 34828788 PMC8624473

[17] Merchant TE Kun LE Wu S Xiong X Sanford RA Boop FA . Phase II trial of conformal radiation therapy for pediatric low-grade glioma. J Clin Oncol. (2009) 27:3598–604. doi: 10.1200/JCO.2008.20.9494 PMC3525947 19581536

[18] Greenberger BA Pulsifer MB Ebb DH MacDonald SM Jones RM Butler WE . Clinical outcomes and late endocrine, neurocognitive, and visual profiles of proton radiation for pediatric low-grade gliomas. Int J Radiat Oncol Biol Phys. (2014) 89:1060–8. doi: 10.1016/j.ijrobp.2014.04.053 25035209

[19] Heitzer AM Kahalley LS Minard CG Stafford C Grosshans DR Okcu MF . Treatment age and neurocognitive outcomes following proton beam radiotherapy for pediatric low- and intermediate-grade gliomas. Pediatr Blood Cancer. (2021) 68:e29096. doi: 10.1002/pbc.29096 34019329 PMC9040342

[20] Indelicato DJ Rotondo RL Uezono H Sandler ES Aldana PR Ranalli NJ . Outcomes following proton therapy for pediatric low-grade glioma. Int J Radiat Oncol Biol Phys. (2019) 104:149–56. doi: 10.1016/j.ijrobp.2019.01.078 30684665

[21] Rodrigues AJ Jin MC Wu A Bhambhvani HP Li G Grant GA . Risk of secondary neoplasms after external-beam radiation therapy treatment of pediatric low-grade gliomas: a SEER analysis, 1973–2015. J Neurosurg Pediatr. (2021) 28(3):306–14. doi: 10.3171/2021.1.PEDS20859 34144522

[22] Ludmir EB Mahajan A Paulino AC Jones JY Ketonen LM Su JM . Increased risk of pseudoprogression among pediatric low-grade glioma patients treated with proton versus photon radiotherapy. Neuro Oncol. (2019) 21:686–95. doi: 10.1093/neuonc/noz042 PMC6502497 30753704

[23] Cherlow JM Shaw DWW Margraf LR Bowers DC Huang J Fouladi M . Conformal radiation therapy for pediatric patients with low-grade glioma: results from the children's oncology group phase 2 study ACNS0221. Int J Radiat Oncol Biol Phys. (2019) 103(4):861–8. doi: 10.1016/j.ijrobp.2018.11.004 PMC6548322 30419305

[24] Mannina EM Bartlett GK McMullen KP . Extended volumetric follow-up of juvenile pilocytic astrocytomas treated with proton beam therapy. Int J Part Ther. (2016) 3(2):291–9. 10.14338/IJPT-16-00020.1 PMC6871611 31772980

[25] Raikar SS Halloran DR Elliot M McHugh M Patel S Gauvain KM . Outcomes of pediatric low-grade gliomas treated with radiation therapy: a single-institution study. J Pediatr Hematol Oncol. (2014) 36(6):e366–70. 10.1097/MPH.0000000000000142 PMC4197833 24714505

[26] Paulino AC Mazloom A Terashima K Su J Adesina AM Okcu MF . Intensity-modulated radiotherapy (IMRT) in pediatric low-grade glioma. Cancer. (2013) 119(14):2654–9. 10.1002/cncr.28118 23633429

[27] Merchant TE Conklin HM Wu S Lustig RH Xiong X . Late effects of conformal radiation therapy for pediatric patients with low-grade glioma: prospective evaluation of cognitive, endocrine, and hearing deficits. J Clin Oncol. (2009) 27:3691–7. doi: 10.1200/JCO.2008.21.2738 PMC2799064 19581535

[28] Marcus KJ Goumnerova L Billett AL Lavally B Scott RM Bishop K . Stereotactic radiotherapy for localized low-grade gliomas in children: final results of a prospective trial. Int J Radiat Oncol Biol Phys. (2005) 61:374–9. doi: 10.1016/j.ijrobp.2004.06.012 15667955

[29] Hug EB Muenter MW Archambeau JO DeVries A Liwnicz B Loredo LN . Conformal proton radiation therapy for pediatric low-grade astrocytomas. Strahlenther Onkol. (2002) 178(1):10–7. 10.1007/s00066-002-0874-2 11977386

[30] Chambrelant I Eber J Antoni D Burckel H Noël G Auvergne R . Proton therapy and gliomas: a systematic review. Radiation. (2021) :218–33. doi: 10.3390/radiation1030019

[31] Kortmann RD Timmermann B Taylor RE Scarzello G Plasswilm L Paulsen F . Current and future strategies in radiotherapy of childhood low-grade glioma of the brain. Part I: Treatment modalities of radiation therapy. Strahlenther Onkol. (2003) 179:509–20. doi: 10.1007/s00066-003-9104-9 14509949

[32] Marta GN Murphy E Chao S Yu JS Suh JH . The incidence of second brain tumors related to cranial irradiation. Expert Rev Anticancer Ther. (2015) 15:295–304. doi: 10.1586/14737140.2015.989839 25482749

[33] Mueller S Sear K Hills NK Chettout N Afghani S Gastelum E . Risk of first and recurrent stroke in childhood cancer survivors treated with cranial and cervical radiation therapy. Int J Radiat Oncol Biol Phys. (2013) 86:643–8. doi: 10.1016/j.ijrobp.2013.03.004 PMC3696632 23623405

[34] Lawrie TA Evans J Gillespie D Erridge S Vale L Kernohan A . Long-term side effects of radiotherapy, with or without chemotherapy, for glioma. Cochrane Database Syst Rev. (2018) 8(8):CD013047. doi: 10.1002/14651858.CD013047 PMC6699681 31425631

[35] Ater JL Zhou T Holmes E Mazewski CM Booth TN Freyer DR . Randomized study of two chemotherapy regimens for treatment of low-grade glioma in young children: a report from the Children's Oncology Group. J Clin Oncol. (2012) 30:2641–7. doi: 10.1200/JCO.2011.36.6054 PMC3413276 22665535

[36] Lu VM Welby JP Laack NN Mahajan A Daniels DJ . Pseudoprogression after radiation therapies for low grade glioma in children and adults: A systematic review and meta-analysis. Radiother Oncol. (2020) 142:36–42. doi: 10.1016/j.radonc.2019.07.013 31431375

[37] Naftel RP Pollack IF Zuccoli G Deutsch M Jakacki RI . Pseudoprogression of low-grade gliomas after radiotherapy. Pediatr Blood Cancer. (2015) 62:35–9. doi: 10.1002/pbc.25179 25213668

[38] Tsang DS Murphy ES Lucas JT Lagiou P Acharya S Merchant TE . Pseudoprogression in pediatric low-grade glioma after irradiation. J Neurooncol. (2017) 135:371–9. doi: 10.1007/s11060-017-2583-9 PMC8717050 28752498

[39] Stock A Hancken CV Kandels D Kortmann RD Dietzsch S Timmermann B . Pseudoprogression is frequent after front-line radiation therapy in pediatric low-grade glioma: results from the german low-grade glioma cohort. Int J Radiat Oncol Biol Phys. (2022) 112:1190–202. doi: 10.1016/j.ijrobp.2021.12.007 34933039

[40] Upadhyay R Yadav D Venkatesulu BP Singh R Baliga S Raval RR . Risk of secondary Malignant neoplasms in children following proton therapy vs. photon therapy for primary CNS tumors: A systematic review and meta-analysis. Front Oncol. (2022) 12:893855. doi: 10.3389/fonc.2022.893855 36033525 PMC9413159

[41] Sharif S Ferner R Birch JM Gillespie JE Gattamaneni HR Baser ME . Second primary tumors in neurofibromatosis 1 patients treated for optic glioma: substantial risks after radiotherapy. J Clin Oncol. (2006) 24:2570–5. doi: 10.1200/JCO.2005.03.8349 16735710

[42] Murphy ES Xie H Merchant TE Yu JS Chao ST Suh JH . Review of cranial radiotherapy-induced vasculopathy. J Neurooncol. (2015) 122:421–9. doi: 10.1007/s11060-015-1732-2 25670390

[43] Bowers DC Mulne AF Reisch JS Elterman RD Munoz L Booth T . Nonperioperative strokes in children with central nervous system tumors. Cancer. (2002) 94:1094–101. doi: 10.1002/cncr.10353.abs 11920480

[44] Bowers DC Liu Y Leisenring W McNeil E Stovall M Gurney JG . Late-occurring stroke among long-term survivors of childhood leukemia and brain tumors: a report from the Childhood Cancer Survivor Study. J Clin Oncol. (2006) 24:5277–82. doi: 10.1200/JCO.2006.07.2884 17088567

[45] Campen CJ Kranick SM Kasner SE Kessler SK Zimmerman RA Lustig R . Cranial irradiation increases risk of stroke in pediatric brain tumor survivors. Stroke. (2012) 43:3035–40. doi: 10.1161/STROKEAHA.112.661561 PMC3492057 22968468

[46] Kortmann RD Timmermann B Taylor RE Scarzello G Plasswilm L Paulsen F . Current and future strategies in radiotherapy of childhood low-grade glioma of the brain. Part II: Treatment-related late toxicity. Strahlenther Onkol. (2003) 179:585–97. doi: 10.1007/s00066-003-8104-0 14628124

[47] Desai SS Paulino AC Mai WY Teh BS . Radiation-induced moyamoya syndrome. Int J Radiat Oncol Biol Phys. (2006) 65:1222–7. doi: 10.1016/j.ijrobp.2006.01.038 16626890

[48] Fouladi M Langston J Mulhern R Jones D Xiong X Yang J . Silent lacunar lesions detected by magnetic resonance imaging of children with brain tumors: a late sequela of therapy. J Clin Oncol. (2000) 18:824–31. doi: 10.1200/JCO.2000.18.4.824 10673524

[49] Koike S Aida N Hata M Fujita K Ozawa Y Inoue T . Asymptomatic radiation-induced telangiectasia in children after cranial irradiation: frequency, latency, and dose relation. Radiology. (2004) 230:93–9. doi: 10.1016/j.ccell.2020.03.011. 14645879

[50] Ullrich NJ Robertson R Kinnamon DD Scott RM Kieran MW Turner CD . Moyamoya following cranial irradiation for primary brain tumors in children. Neurology. (2007) 68:932–8. doi: 10.1212/01.wnl.0000257095.33125.48 17372129

[51] Cella D Chang CH Lai JS Webster K . Advances in quality of life measurements in oncology patients. Semin Oncol. (2002) 29:60–8. doi: 10.1016/S0093-7754(02)70176-0 12082656

[52] Heimans JJ Taphoorn MJ . Impact of brain tumour treatment on quality of life. J Neurol. (2002) 249:955–60. doi: 10.1007/s00415-002-0839-5 12195437

[53] Liu R Page M Solheim K Fox S Chang SM . Quality of life in adults with brain tumors: current knowledge and future directions. Neuro Oncol. (2009) 11:330–9. doi: 10.1215/15228517-2008-093 PMC2718978 19001097

[54] Armstrong GT Liu Q Yasui Y Huang S Ness KK Leisenring W . Long-term outcomes among adult survivors of childhood central nervous system Malignancies in the Childhood Cancer Survivor Study. J Natl Cancer Inst. (2009) 101:946–58. doi: 10.1093/jnci/djp148 PMC2704230 19535780

[55] Gupta P Jalali R . Long-term survivors of childhood brain tumors: impact on general health and quality of life. Curr Neurol Neurosci Rep. (2017) 17:99. doi: 10.1007/s11910-017-0808-0 29119343

[56] Kun LE Mulhern RK Crisco JJ . Quality of life in children treated for brain tumors. Intellectual, emotional, and academic function. J Neurosurg. (1983) 58:1–6. doi: 10.3171/jns.1983.58.1.0001 6847894

[57] Macartney G Harrison MB VanDenKerkhof E Stacey D McCarthy P . Quality of life and symptoms in pediatric brain tumor survivors: a systematic review. J Pediatr Oncol Nurs. (2014) 31:65–77. doi: 10.1177/1043454213520191 24608699

[58] King S Exley J Parks S Ball S Bienkowska-Gibbs T MacLure C . The use and impact of quality of life assessment tools in clinical care settings for cancer patients, with a particular emphasis on brain cancer: insights from a systematic review and stakeholder consultations. Qual Life Res. (2016) 25:2245–56. doi: 10.1007/s11136-016-1278-6 PMC4980409 27039304

[59] Eiser C Vance YH Horne B Glaser A Galvin H . The value of the PedsQLTM in assessing quality of life in survivors of childhood cancer. Child Care Health Dev. (2003) 29:95–102. doi: 10.1046/j.1365-2214.2003.00318.x 12603354

[60] Palmer SN Meeske KA Katz ER Burwinkle TM Varni JW . The PedsQL Brain Tumor Module: initial reliability and validity. Pediatr Blood Cancer. (2007) 49:287–93. doi: 10.1002/pbc.21026 16991131

